# A Putative Mechanism of Age-Related Synaptic Dysfunction Based on the Impact of IGF-1 Receptor Signaling on Synaptic CaMKIIα Phosphorylation

**DOI:** 10.3389/fnana.2018.00035

**Published:** 2018-05-14

**Authors:** Olalekan M. Ogundele, Joaquin Pardo, Joseph Francis, Rodolfo G. Goya, Charles C. Lee

**Affiliations:** ^1^Department of Comparative Biomedical Sciences, School of Veterinary Medicine, Louisiana State University, Baton Rouge, LA, United States; ^2^Institute for Biochemical Research of La Plata, School of Medicine, National University of La Plata, La Plata, Argentina

**Keywords:** IGF-1/IGF-1R, aging, CaMKIIα, MAPK/ErK, KCa2.2

## Abstract

Insulin-like growth factor 1 receptor (IGF-1R) signaling regulates the activity and phosphorylation of downstream kinases linked to inflammation, neurodevelopment, aging and synaptic function. In addition to the control of Ca^2+^ currents, IGF-1R signaling modulates the activity of calcium-calmodulin-dependent kinase 2 alpha (CaMKIIα) and mitogen activated protein kinase (MAPK/ErK) through multiple signaling pathways. These proteins (CaMKIIα and MAPK) regulate Ca^2+^ movement and long-term potentiation (LTP). Since IGF-1R controls the synaptic activity of Ca^2+^, CaMKIIα and MAPK signaling, the possible mechanism through which an age-dependent change in IGF-1R can alter the synaptic expression and phosphorylation of these proteins in aging needs to be investigated. In this study, we evaluated the relationship between an age-dependent change in brain IGF-1R and phosphorylation of CaMKIIα/MAPK. Furthermore, we elucidated possible mechanisms through which dysregulated CaMKIIα/MAPK interaction may be linked to a change in neurotransmitter processing and synaptic function. Male C57BL/6 *VGAT-Venus* mice at postnatal days 80 (P80), 365 and 730 were used to study age-related neural changes in two brain regions associated with cognitive function: hippocampus and prefrontal cortex (PFC). By means of high throughput confocal imaging and quantitative immunoblotting, we evaluated the distribution and expression of IGF-1, IGF-1R, CaMKIIα, p-CaMKIIα, MAPK and p-MAPK in whole brain lysate, hippocampus and cortex. Furthermore, we compared protein expression patterns and regional changes at P80, P365 and P730. Ultimately, we determined the relative phosphorylation pattern of CaMKIIα and MAPK through quantification of neural p-CaMKIIα and p-MAPK/ErK, and IGF-1R expression for P80, P365 and P730 brain samples. In addition to a change in synaptic function, our results show a decrease in neural IGF-1/IGF-1R expression in whole brain, hippocampus and cortex of aged mice. This was associated with a significant upregulation of phosphorylated neural MAPK (p-MAPK) and decrease in total brain CaMKIIα (i.e., CaMKIIα and p-CaMKIIα) in the aged brain. Taken together, we showed that brain aging is associated with a change in neural IGF-1/IGF-1R expression and may be linked to a change in phosphorylation of synaptic kinases (CaMKIIα and MAPK) that are involved in the modulation of LTP.

## Introduction

Brain aging has been implicated in the cause and progression of disease conditions, characterized in part by memory deficits (Reagh and Yassa, 2017; Seeley, 2017), notably in disorders like Alzheimer’s disease (Heckman et al., 2017; Vemuri et al., 2017; Caballero et al., 2018). One underlying cause of the behavioral changes associated with normal or disease-related aging is modifications to synaptic morphology and molecular composition (Bertoni-Freddari et al., 1988, 1992, 1993), which often lead to neuronal cell death, oxidative stress and cytoskeletal defects, which are implicated in age-linked disorders (Wilson et al., 2016, 2017; Pellegrini et al., 2017; Salvadores et al., 2017).

Neurotrophic factors and receptors have important roles during development and in the adult brain. During development, neural progenitor cells develop cytoskeletal structures (neurites) required for cell migration in the developing brain (Chou and Wang, 2016; Hanamura, 2017). In the absence of neurotrophic factors, neuronal development is impaired (Sanford et al., 2008; Park and Poo, 2013). Depending on the stage of development, depletion of neurotrophins may halt neural cell migration and formation of synapses in several brain circuits (Park and Poo, 2013; Chou and Wang, 2016). Therefore, the process of neural circuit formation is a set of molecular events governed by neurotrophic activation of neurotropin receptors (Mousa and Bakhiet, 2013; Bertrand, 2017). Similarly, in the adult nervous system, neurotrophic factors and receptors are required for the maintenance of active synapses (Gómez-Palacio-Schjetnan and Escobar, 2008; Ito-Ishida et al., 2008; Garcia et al., 2012; Ivanov, 2014). There are several neurotropic factors in the brain; insulin-like growth factor-1 (IGF-1), nerve growth factor (NGF) and, brain derived neurotropic factor (BDNF) among others (Yuen et al., 1996; Park and Poo, 2013; Song et al., 2017; Zegarra-Valdivia, 2017), which have associated receptors, such as Insulin-like growth factor 1 receptor (IGF-1R), IGF-1 receptor 2 (IGF-2R), and Tyrosine kinase receptors (RTkA and RTkB; Mousa and Bakhiet, 2013; Dyer et al., 2016; Bertrand, 2017).

IGF-1 and IGF-1R are particularly important because of their role in neurodevelopment, synaptic function and aging (Bartke et al., 2003; Sonntag et al., 2005; Chiu and Cline, 2010; Dyer et al., 2016). As such, changes in their expression pattern have been implicated in the pathophysiology of developmental and age-related neuropsychiatric disorders (Deak and Sonntag, 2012; Green et al., 2014; Dyer et al., 2016). In addition to their involvement in formation of synapses, IGF-1 and IGF-1R act to maintain synapses in the adult brain (Chiu and Cline, 2010; Gazit et al., 2016; Nieto-Estévez et al., 2016; Decourtye et al., 2017; Reim and Schmeisser, 2017). Notably, IGF-1/IGF-1R signaling may alter the activity of proteins directly involved in synaptic plasticity, cognitive and memory function (Bartke et al., 2003; Sonntag et al., 2005; Deak and Sonntag, 2012).

The role of IGF-1—and other neurotropic factors such as BDNF—in neuronal development and synaptic plasticity has been described extensively (Nieto-Estévez et al., 2016; Reim and Schmeisser, 2017). However, a recent study demonstrated that IGF-1R is directly involved in the regulation of presynaptic Ca^2+^ release during long-term potentiation (LTP) in the hippocampus (Gazit et al., 2016). Therefore, both IGF-1 and IGF-1R can directly modulate specific aspects of cognition and memory function in the hippocampus (Sonntag et al., 2005; Deak and Sonntag, 2012). IGF-1-mediated activation of neurotropin receptors, and IGF-1R activation (by IGF-1 or insulin) involves signaling of downstream proteins (Hiney et al., 2009; Liu et al., 2015; Law et al., 2017). These kinases are involved in several pathways associated with synaptic function, growth, inflammation and metabolism (Schumacher et al., 1991; Mynarcik et al., 1997; Siddle, 2011; Fernandez and Torres-Alemán, 2012).

IGF-1 activation of insulin receptor or IGF-1R can initiate Ras/ErK signaling (Lopaczynski, 1999; Moelling et al., 2002; Dyer et al., 2016). Furthermore, Ras/Raf signaling can modulate the phosphorylation of synaptic regulatory calcium-calmodulin-dependent kinase 2 alpha (CaMKIIα; Villalonga et al., 2001; Illario et al., 2003; Wu et al., 2011; DiBattista et al., 2015). Both mitogen activated protein kinase (MAPK/ErK) and CaMKIIα are likely colocalized at synaptic densities (Giovannini et al., 2001; Tsui et al., 2005). As such, the phosphorylation status of these proteins may alter hippocampal LTP and depression (LTD; Giovannini et al., 2001; Derkach et al., 2007). Therefore, a change in IGF-1/IGF-1R may affect synaptic function by altering the balance between synaptic MAPK/ErK and CaMKIIα activity.

CaMKIIα and MAPK/ErK act downstream of IGF-1/IGF-1R in various signaling pathways already described in neurons (Chiu and Cline, 2010; Song et al., 2010; Zuloaga et al., 2013). CaMKIIα controls LTP by regulating ionotropic receptors and ion movement at post-synaptic densities (PSDs; Wang and Kelly, 2001; Hinds et al., 2003; Mao et al., 2014). Since MAPK/ErK is co-localized with CaMKIIα at PSDs, it can alter the synaptic activity of CaMKIIα by increased phosphorylation (Giovannini et al., 2001; Tsui et al., 2005; Derkach et al., 2007). Physiologically, the LTP process is associated with a synchronous oscillation of Ca^2+^ and K^+^ ions (Bacci et al., 1999; Power et al., 2002; Allen et al., 2011). During LTP, CaMKIIα increase Ca^2+^ currents from inotropic glutamate receptor activation (Sanz-Clemente et al., 2013; Mao et al., 2014; DiBattista et al., 2015) and inhibits small ion conductance channels, such KCa2.2 (Hammond et al., 2006; Lin et al., 2010; Griffith et al., 2016). Conversely, MAPK/ErK can inhibit CaMKIIα (Giovannini et al., 2001), while activating the pore forming sub-units of calcium-activated potassium (KCa2.2) channels (Schrader et al., 2006; Turner and Shieh, 2006).

KCa2.2 channels generates prolonged low-tone K^+^ currents during LTP (Kim and Hoffman, 2008) and the hyperpolarization phase of the action potential (Power et al., 2002; Hammond et al., 2006; Lin et al., 2010). An increase in KCa2.2 activity reduces the threshold of the action potential due to a sustained after-hyperpolarization effect (Power et al., 2002; Stocker, 2004; Stocker et al., 2004; Lin et al., 2010). Thus, age-linked neural changes, which promotes loss of CaMKIIα function, can upregulate KCa2.2 activity through disinhibition of this channel. Additionally, age-dependent increase in Ras/ErK activation can promote KCa2.2 activity by attenuating (phosphorylating) CaMKIIα-linked inhibition of KCa2.2. Furthermore, Ras-ErK signaling can activate (phosphorylate) pore forming subunits of KCa2.2.

**Figure 1:**
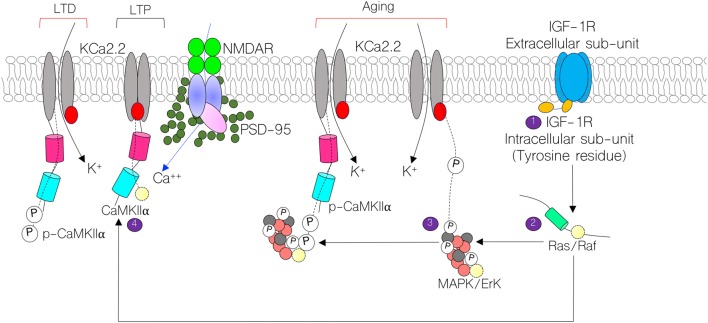
Schematic illustration of the mechanism through which calcium-calmodulin-dependent kinase 2 alpha (CaMKIIα) regulates long-term potentiation (LTP) by modulating KCa2.2 and NMDAR function at post-synaptic densities (PSDs). Here, we show possible contributions of Insulin-like growth factor 1 receptor (IGF-1R; 1,2) signaling in the regulation of KCa2.2 and CaMKIIα. In normal synaptic function, CaMKIIα inhibits the conductance capability of KCa2.2 by blocking calcium binding sites on its intracellular domain. Conversely, in long-term depression (LTD), CaMKIIα is phosphorylated and allows for conductance of K^+^ ions, thereby obliterating synaptic potentials (3,4). In aging, we hypothesize that a change in IGF-1R signaling may reduce synaptic CaMKIIα function by upregulating mitogen activated protein kinase (MAPK/ErK) activity. Thus, increased MAPK/ErK can hyperphosphorylate CaMKIIα (inactivate) and phosphorylate (activate) pore forming subunits of KCa2.2.

Therefore, we asked whether an age-related change in IGF-1/IGF-1R axis is related to changes in synaptic function through age-related alterations of MAPK/ErK/CaMKIIα and KCa2.2 in the hippocampus and PFC (Figure 1)? A decline in IGF-1 and IGF-1R expression has been described in age-related neuropsychiatric and degenerative diseases (Carro et al., 2002; Yaghmaie et al., 2006; Piriz et al., 2011; Puche and Castilla-Cortázar, 2012; Green et al., 2014; Werner and LeRoith, 2014). Therefore, in this study we assessed the differential expression of these synaptic kinases (i.e MAPK/ErK and CaMKIIα) with age in the hippocampus and medial prefrontal cortex (mPFC). Additionally, we examined the relationship between age-related change in neural MAPK/ErK/CaMKIIα activity and expression of synaptic markers—neurotransmitter transporters—in the hippocampus (CA1) and mPFC.

## Materials and Methods

### Animal Strain

Male C57BL/6 *VGAT-Venus* mice of the following age groups were used for this study; postnatal days 80 (P80; young adult; *n* = 11), P365 (middle aged; *n* = 10), P730 (elderly; *n* = 8). The vesicular GABA transporter (VGAT) Venus mice have been previously developed and characterized by Wang et al. (2009). The animals (i.e., VGAT-Venus) used to establish the colony for this study were obtained from Dr. Janice Nagle at Wesleyan University and bred at the vivarium of the Louisiana State University School of Veterinary Medicine. VGAT-Venus mice are transgenic mice bred on a C57BL/6J background and carry no mutations or abnormalities. These mice express a fluorescence protein called Venus (a modified yellow fluorescence protein developed by Atsushi Miyawaki at RIKEN, Wako, Japan) in inhibitory GABAergic and Glycinergic neurons (Wang et al., 2009). VGAT-Venus mice were used because it enables a simple method for assaying changes in inhibitory neuronal composition through immunofluorescence imaging (Lee et al., 2015).

All animals used for this experiment weighed between 22–27 grams. Animals were kept under standard laboratory conditions and handled in accordance to NIH guidelines for animal care and use in research. All protocols used were reviewed and approved by the Institutional Animal Care and Use Committee of the Louisiana State University School of Veterinary Medicine.

### Sample Preparation

After the brains were collected, the right and left hemisphere were used for immunofluorescence or immunoblotting preparations, respectively.

### Immunofluorescence

Animals were deeply anesthetized via inhalation of isoflurane in an enclosed chamber, then perfused transcardially through the left ventricle using 10 mM phosphate buffered saline (PBS). The right half of the brain was collected and rapidly fixed in 4% PB paraformaldehyde (PFA) overnight at 4°C. Subsequently, the fixed brain samples were transferred into 4% PB-PFA containing 30% sucrose for cryopreservation. Cryopreservation was performed at 4°C for 72 h. Free-floating cryostat sections (20 μm thick) were obtained using a Leica Cryostat and collected in 10 mM PBS at 4°C. The sections were washed three times (5 min each) in 10 mM PBS (*pH 7.4*) on a tissue rocker. Blocking was done in normal goat serum (Vector Labs), prepared in 10 mM PBS+0.03% Triton-X 100, for 2 h at room temperature. The sections were incubated in primary antibody solution overnight at 4°C [Rabbit anti-IGF-1R (1:100; ThermoScientific-MA5-15148), Mouse anti SK2.2 (EMD Millipore Q2650573; *1:250*), Rabbit anti-MAPK/ErK1/ErK2 (*1:100*; Cell Signaling-#9102) and Mouse anti- CaMKIIα (*1:100*; Cell Signaling-#50049)]. The primary antibodies were diluted appropriately in 10 mM PBS, 0.03% Triton-X 100 and normal goat serum. It is important to note that VGAT-Venus mice express Venus in inhibitory GABAergic and Glycinergic neurons: therefore, no staining was necessary for Venus fluorescence observation. Subsequently, the sections were washed as previously described and incubated in secondary antibody solution [Goat anti Rabbit 568, Goat anti Rabbit 594 and Goat anti Mouse 568 (diluted at *1:1000*) prepared in 10 mM PBS, 0.03% Triton X-100 and Normal Goat Serum] at room temperature (1 h). Immunolabeled sections were washed and mounted on gelatin-coated slides using a plain or DAPI containing anti-fade mounting medium (Vector Labs).

### Confocal Microscopy

Imaging of immunolabeled proteins in the hippocampus and cortex was performed by confocal microscopy (Olympus FluoView 10i). Fluorescence intensity was estimated for CaMKIIα, IGF-1R, MAPK/ErK and KCa2.2 using ImageJ (Burgess et al., 2010; McCloy et al., 2014). In addition, cell counting was conducted to determine the distribution of Venus-expressing neurons per unit area in the hippocampus (CA1-DG field) and mPFC (Layer V) using ImageJ (Grishagin, 2015). Fluorescence quantification and cell counting was conducted in *n* = 10 fields for *n* = 6 consecutive brain (serial) sections per animal. The average fluorescence intensity and cell count was determined and compared for all groups in One-Way analysis of variance (ANOVA) with Tukey *post hoc* test. Statistical analysis was performed in GraphPad Prism Version 7.0.

### Immunoblotting

The left whole brain was rapidly frozen and homogenized using a low speed hand-held homogenizer. The brain homogenate was centrifuged at 12,500 g for 15 min (4°C) to isolate whole brain lysate. Fifteen microgram of protein (3 biological replicates for all samples), obtained from brain tissue homogenate, was processed per well. After western blotting, protein was detected using the following primary antibodies; Rabbit anti IGF-1R (Cell Signaling-#3027s), Mouse anti-IGF-1 (abcam-# ab176523), Rabbit anti-MAPK/ErK1/ErK2 (1:100; Cell Signaling-#9102), Rabbit anti-phospho-MAPK/ErK1/ErK2 (Cell Signaling-#4370s; sites: Thr202/Tyr204), Mouse anti-CaMKIIα (Cell Signaling-#50049), Rabbit anti-phospho-CaMKIIα (Cell Signaling-#12716s; site: Thr286), Mouse anti SK2.2 (EMD Millipore-#Q2650573), Rabbit anti PSD-95 (Cell Signaling-#3450s), Rabbit anti Homer-1 (Proteintech-#124-33-1-AP), Rabbit anti Synaptophysin (Cell Signaling-#5461s), Rabbit anti vesicular glutamate transporter 2 (VGLUT2; abcam-#ab84103), Rabbit anti GAPDH (Cell Signaling-#5174s). Subsequently, the primary antibodies were detected with HRP-conjugated Goat anti Rabbit (Invitrogen #65-6120) and Goat anti Mouse (Invitrogen #65-6520) secondary antibodies following which the reaction was developed using a chemiluminescence substrate (Thermofisher-#34579). Protein expression was quantified and normalized with the housekeeping protein (GAPDH) and synaptic proteins (PSD-95, Homer1 and Synaptophysin) expression using *Image Lab version 5.2.1* (BioRad, Hercules, CA, USA). Multiple controls were used for normalizing each protein of interest because of a general decline in neural proteins with age (Carney et al., 1991; Schimanski and Barnes, 2010). Subsequently, normalized protein expression data was analyzed through *One-Way* ANOVA (with Tukey *Post hoc* test) in GraphPad Prism Version 7.0. The outcome was presented as bar chart with error bars representing the mean ± SEM respectively.

## Statistics

Analysis was conducted with the GraphPad Prism Version 7.0. For the immunofluorescence results, the average fluorescence intensity and cell count was determined and compared for all groups in One-Way ANOVA with Tukey *post hoc* test. For immunoblotting protein expression data, *One-Way* ANOVA (with Tukey *Post hoc* test) was performed. The outcomes are presented as bar chart with error bars representing the mean ± SEM respectively.

## Results

### Changes in Control Protein Expression With Age

We observed a significant decrease in expression level of control proteins—GAPDH, PSD-95 and Homer1—in brain lysate prepared from aged mice (P730) when compared with P80 and P365 brain samples. This may have resulted from age-related decrease in neural protein synthesis, or loss of protein due to oxidation (Carney et al., 1991; Schimanski and Barnes, 2010). To ascertain an age-linked protein depletion, equal volume (20 μl) and proteins concentration (15 μg/well) were examined via Western blot for each sample (animal) across all groups. As such, P730 mice exhibited a significant decrease in MAPK, CaMKIIα, p-CaMKIIα, IGF-1 and IGF-1R. The results were normalized by the corresponding expression of control proteins (GAPDH, Homer1, PSD-95 and synaptophysin) from the same sample in multiple trials. Interestingly, control proteins—GAPDH, Homer1 and PSD-95–were reduced significantly in P730 brain samples. Therefore, band intensity for a protein of interest was divided by band intensity for control proteins for the same sample. Ultimately, the average expression after normalizing with various control proteins was adopted as the normalized expression for the protein. However, not all proteins were reduced in the aged brain. *Synaptophysin* (a control) and *p-MAPK/ErK* were significantly upregulated in P730 brain lysates when compared with P80 and P365 samples. Therefore, a decrease in a control protein does not connote a decrease in protein sample loaded for the P730 group.

### Age-Dependent Change in Neural IGF-1 and IGF-1R Expression

**Figure 2:**
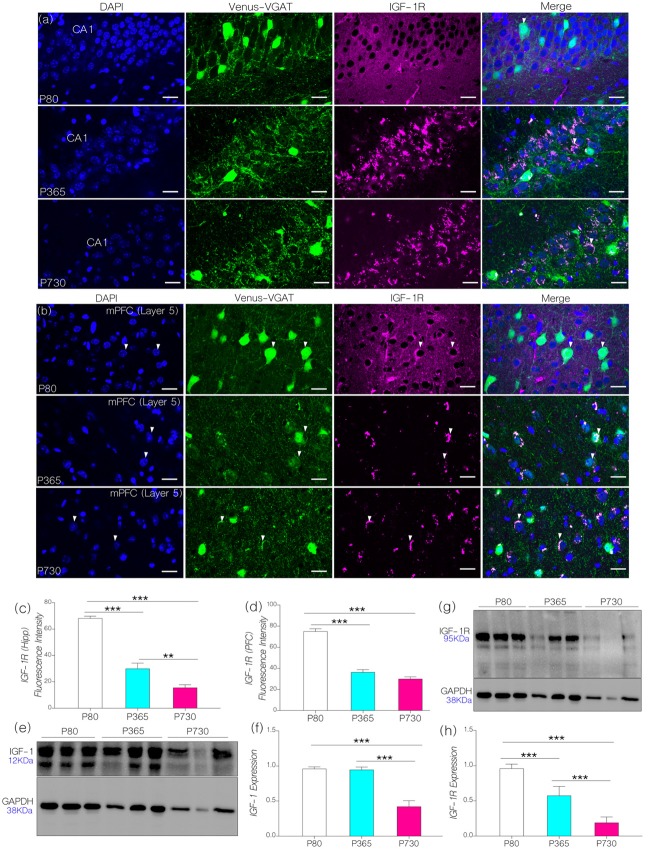
**(A,B)** Representative confocal images showing the distribution of IGF-1R hippocampus and medial prefrontal cortex (mPFC) of postnatal days 80 (P80), P365 and P730 mice (scale bar = 20 μm). Arrow heads show the distribution of IGF-1R in Layer V vesicular GABA transporter (VGAT) positive neurons. **(C)** Bar chart (One-Way analyses of variance (ANOVA)) showing a decrease in hippocampal IGF-1R expression at P365 (*p* < 0.001) when compared with P80 hippocampus. There was further decline in hippocampal IGF-1R at P730 (*p* < 0.01) when compared with P365, and P80 (*p* < 0.001) hippocampus.** (D)** Bar chart (One-Way ANOVA) showing a significant decrease in IGF-1R in the mPFC of P365 and P730 mice when compared with P80 (*p* < 0.001). No significant change was seen for hippocampal IGF-1R expression when we compared P365 and P730 hippocampus. **(E,F)** Quantitative western blots showing a significant decrease in insulin-like growth factor-1 (IGF-1) expression in total brain lysate at P730 when compared with P80 (*p* < 0.001) and P365 (*p* < 0.001). No significant change in IGF-1 was observed at P365 when compared with P80. Protein expression per lane was normalized with GAPDH in 15 μg total protein for P80, P365 and P730 groups. **(G,H)** IGF-1R expression reduced in total brain lysate at P365 (*p* < 0.001) when compared with P80. There was a further decrease in neural IGF-1R expression at P730 (*p* < 0.001) when compared with P80 and P365. **p < 0.01, ***p < 0.001.

First, we evaluated the distribution of IGF-1R in immunolabeled brain sections containing the cortex and hippocampus. IGF-1R expression was estimated through quantification of fluorescence (see “Materials and Methods” section). For this procedure, a constant exposure time and contrast was adopted for all sections, relative to background staining. We observed an age-dependent decrease in hippocampal IGF-1R expression when P365 (*p* < 0.001) and P730 (*p* < 0.001) mice were compared with their P80 counterparts (Figures 2A,C). In addition, there was a significant reduction of IGF-1R staining in the mPFC at P365 (*p* < 0.001) and P730 (*p* < 0.001) when compared with the P80 mice (Figures 2B,D).

In quantitative immunoblotting of whole brain lysate, there was an age-dependent decrease in neural IGF-1 level when P730 mice were compared with P80 and P365 mice (Figures 2E,F; *p* < 0.001). Interestingly, there was no significant change in neural IGF-1 level at P365 when compared with P80 IGF-1 expression. Similar to the observations from confocal quantification, there was a significant decrease (*p* < 0.001) in IGF-1R expression with age in whole brain lysate of P365 and P730 mice (Figures 2E,F). Furthermore, a significant decline (*p* < 0.001) was observed when comparing P730 with P365 neural IGF-1R expression (Figures 2G,H). Based on these outcomes, we deduced that a decline in both IGF-1 and IGF-1R are associated with aging in the hippocampus and cortex. While IGF-1 decline occurred later (P730) than IGF-1R depletion in the hippocampus and cortex (P365). This outcome is based on the age ranges adopted for this study. P80 expression was used as a baseline for immunofluorescence and immunoblotting analysis.

### Phosphorylation of Neural MAPK/ErK Increased With Age

**Figure 3:**
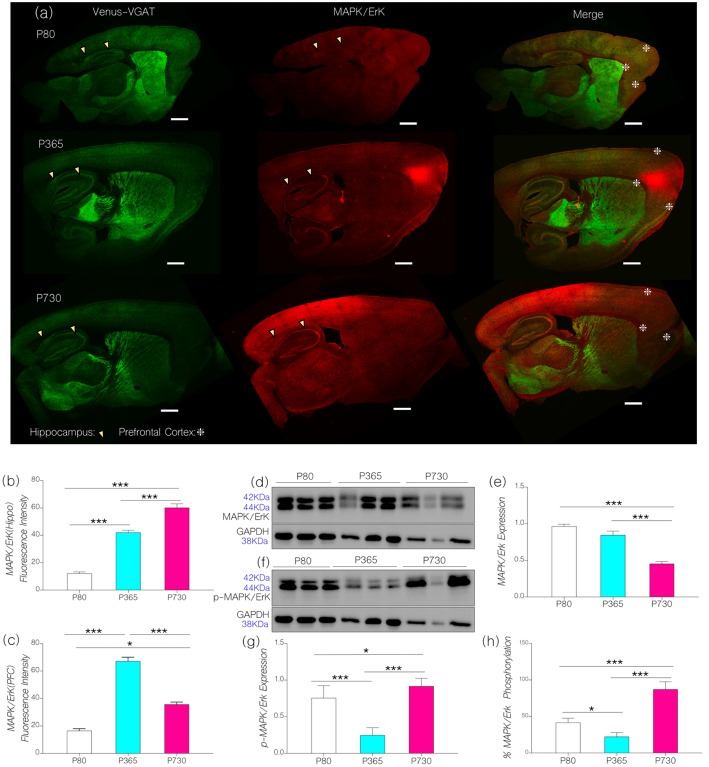
**(A)** Representative confocal images showing the distribution of MAPK/ErK in the cortex (mPFC) and hippocampus (arrow heads indicates CA1) of P80. P365 and P730 mice (scale bar = 100 μm). Arrowheads indicate the CA1 area of the hippocampus. Star signs depict the margins of the PFC. **(B)** Bar chart showing an increase in hippocampal MAPK/ErK expression for P365 (*p* < 0.001) and P730 (*p* < 0.001) groups when compared with P80. **(C)** Bar chart showing an increase in the expression of MAPK/ErK in the mPFC of P365 mice. MAPK/ErK expression was higher at P365 when compared with P80 (*p* < 0.001) and P730 (*p* < 0.001). Equally, MAPK/ErK expression increased in the mPFC of P730 mice when compared with P80 (*p* < 0.05). **(D)** Immunoblots showing the expression of MAPK/ErK in total brain lysate for P80, P365 and P730 groups. **(E)** Bar chart showing a decrease in normalized expression of MAPK/ErK in the brain of P730 mice. **(F–H)** Quantitative immunoblots showing a change in the expression of phosphorylated MAPK/ErK at P365 and P730. Percentage p-MAPK/ErK increased significantly in the P730 (85%; *p* < 0.001) group when compared with P80 (47%) and P365 (25%). *p < 0.05, ***p < 0.001.

Using confocal imaging and quantification techniques, we estimated and normalized the expression of MAPK/ErK (fluorescence) in whole brain sagittal sections (Figure 3A). In P365 vs. P80 mice, we recorded a significant increase in prefrontal cortical and hippocampal MAPK/ErK expression (Figure 3B; *p* < 0.001). At P730, MAPK/ErK expression varied between the hippocampus and mPFC (Figure 3C). Hippocampal MAPK/ErK expression increased at P730 when compared with P365 (*p* < 0.001) and P80 (*p* < 0.001), but reduced significantly in the mPFC when P730 mice were compared with P365 (Figure 3C; *p* < 0.001). However, mPFC MAPK/ErK expression at P730 was higher than the baseline (vs. P80; *p* < 0.05). Therefore, based on our hypothesis, a change in neural IGF-1/IGF-1R expression may be associated with the region-specific change in MAPK/ErK expression. As such, a reduction in hippocampal and mPFC IGF-1R expression was accompanied by an increase in MAPK/ErK.

Since MAPK/ErK is active in its phosphorylated form (Ferrer et al., 2001; Hoofnagle et al., 2004), we compared the distribution of MAPK/ErK and phosphorylated MAPK/ErK in whole brain lysates from P80, P365 and P730 mice. Subsequently, we determined the percentage phosphorylation of neural MAPK/ErK by comparing GAPDH-normalized expression of MAPK/ErK and p-MAPK/ErK in whole brain lysates [p-MAPK/(p-MAPK+MAPK) × 100]. At P365, there was no significant change in neural MAPK/ErK expression when compared with P80 (baseline) MAPK/ErK expression (Figures 3D,E). However, total brain MAPK/ErK expression reduced significantly at P730 (Figures 3D,E; *p* < 0.001). In subsequent analysis, we found age-related differences in non-phosphorylated to phosphorylated forms of MAPK/ErK (Figures 3F,G). As such, in P730 mice there was a significant increase in p-MAPK/ErK in total brain lysate when compared with P80 (*p* < 0.05) and P365 (*p* < 0.001). Based on these outcomes, the percentage of normalized phosphorylated MAPK/ErK was *85%* for P730 mice when compared with P80 (*47%*) and P365 (*25%*; Figure 3H).

From these outcomes, we deduce that a change in IGF-1/IGF-1R signaling may be linked with an increased conversion of MAPK/ErK to p-MAPK/ErK in the brain of aged mice. It is important to note that immunoblot outcomes for protein expression gives brain-specific expression, while confocal imaging depicts region specific (mPFC: *Layer V* and CA1) expression. Although total brain MAPK/ErK did not change at P365 (Figure 3E), mPFC and CA1 MAPK/ErK expressions increased significantly when compared with P80 (Figures 3B,C). Likewise, in spite of a decrease in brain MAPK/ErK at P730 (Figure 3E), hippocampal and cortical distribution of the proteins were significantly higher than what was recorded at P80 (Figures 3B,C). Furthermore, mPFC expression reduced (*p* < 0.001; Figure 3C), while CA1 expression increased, significantly at P730 (*p* < 0.001) when compared with P365 levels.

### Age-Linked Depletion of Brain CaMKIIα

**Figure 4:**
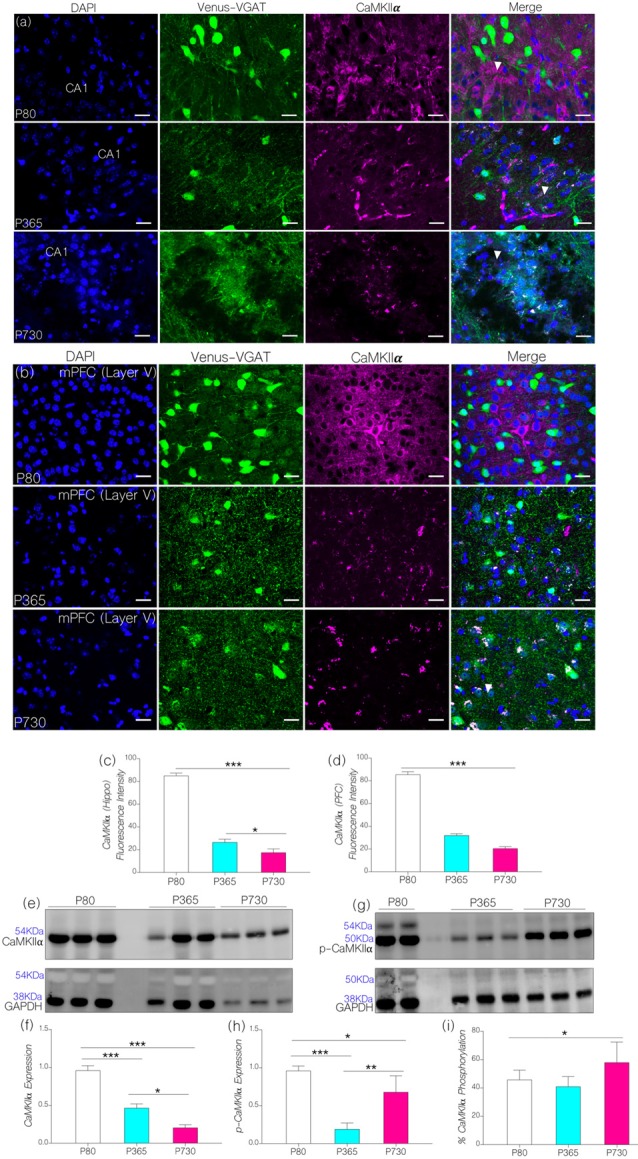
**(A,B)** Representative confocal images showing a substantive decrease in cortical and hippocampal CaMKIIα expression for the P365 and P730 groups (scale bar = 100 μm). **(C)** Bar chart showing a significant decrease in hippocampal CaMKIIα expression for P365 (*p* < 0.001) and P730 (*p* < 0.001) mice when compared with P80 expression level. **(D)** Bar chart showing a decrease in CaMKIIα expression for the mPFC (arrow head) for P365 (*p* < 0.001) and P730 mice (*p* < 0.001) when compared with P80. **(E,F)** Quantitative western blots showing a decrease in CaMKIIα in total brain lysate for P365 (*p* < 0.001) and P730 (*p* < 0.001) mice when compared with the P80 group. A significant decrease was also seen between P365 and P730 (*p* < 0.05). **(G,H)** Immunoblots showing a decrease in brain p-CaMKIIα for P365 (*p* < 0.001) and P730 (*p* < 0.05) mice when compared with P80 p-CaMKIIα expression. An increase in p-CaMKIIα was observed when P730 was compared with P365 (*p* < 0.01). **(I)** Percentage phosphorylated CaMKIIα expression increased significantly at P730 (*p* < 0.05) when compared with P80 and P365. *p < 0.05, **p < 0.01, ***p < 0.001.

Based on our hypothesis (Figure 1), an increase in the expression of brain p-MAPK/ErK may alter synaptic CaMKIIα function through phosphorylation (inactivation). In support of this proposition, an increase in the percentage of brain p-MAPK/ErK was associated with a significant decrease in CA1 and mPFC CaMKIIα expression with age (Figures 4A,B). Normalized fluorescence intensity for immunolabeled CaMKIIα reduced significantly in the hippocampus at P365 (*p* < 0.001) and P730 (*p* < 0.001) when compared with the control (Figure 4C). Likewise, there was a significant reduction in prefrontal cortical expression of CaMKIIα for P365 and P730 mice when compared with P80 (*p* < 0.001; Figure 4D). The outcome for confocal fluorescence quantification was further confirmed through immunoblot quantification of CaMKIIα in whole brain lysate. As such CaMKIIα expression reduced significantly in the lysate prepared from P365 and P730 mice brains when compared with P80 brain lysate in immunoblotting (Figures 4E,F; *p* < 0.001). A further decline in brain CaMKIIα was observed at P730; when compared with P365 CaMKIIα expression (*p* < 0.05; Figure 4F).

Since p-MAPK/ErK can phosphorylate synaptic CaMKIIα (Giovannini et al., 2001; Tsui et al., 2005), we evaluated the significance of increased p-MAPK/ErK (P730) on the normalized expression of p-CaMKIIα in whole brain lysate. In addition to a decrease in CaMKIIα, P365 and P730 mice exhibited a significant depletion of neural p-CaMKIIα when compared with P80 expression (*p* < 0.001 and *p* < 0.05 respectively; Figures 4G,H). CaMKIIα expression decreased significantly in P730 brain lysate when compared with P365 expression (*p* < 0.05; Figures 4E,F). Conversely, p-CaMKIIα expression increased significantly in P730 brain when compared with P365 expression (*p* < 0.01; Figures 4G,H). In subsequent analysis, we determined percentage phosphorylation of CaMKIIα by comparing normalized expression of CaMKIIα and p-CaMKIIα for P80, P365 and P730 brain lysates [p-CaMKIIα/(p-CaMKIIα+CaMKIIα) × 100] (Figure 4I). Interestingly, there was a significant increase in percentage phosphorylated CaMKIIα at P730 (*p* < 0.05) when compared with P80 and P365 groups. Taken together, our results show a significant decrease in total CaMKIIα (*p* < 0.05) at P365. However, for P730 brain, percentage phosphorylated CaMKIIα was upregulated in addition to a decrease in neural CaMKIIα expression (Figures 4F–I).

### Differential Expression of Small-Ion Conductance Channels KCa2.2 in the Hippocampus-PFC Axis

We hypothesized that CaMKIIα regulation of KCa2.2 may be altered because of a decrease in neural CaMKIIα expression (P356 and P730) and increased percentage of phosphorylated CaMKIIα in the P730 brain (Figures 4E–I). Moreover, in addition to increased CaMKIIα phosphorylation by p-MAPK/ErK, the protein (i.e., p-MAPK/ErK) may directly activate the pore forming subunit of KCa2.2 (Figure 1). Owing to a decrease in CaMKIIα expression or an increased CaMKIIα phosphorylation, the activity of KCa2.2 may become upregulated. Consequently, either downregulation of CaMKIIα or an increased p-MAPK/ErK-mediated KCa2.2 phosphorylation would lead to attenuation of synaptic potentials. This may be related to prolonged after-hyperpolarization currents that are linked to increased KCa2.2 activity at synapses (K^+^ ion movement; Figure 1).

**Figure 5:**
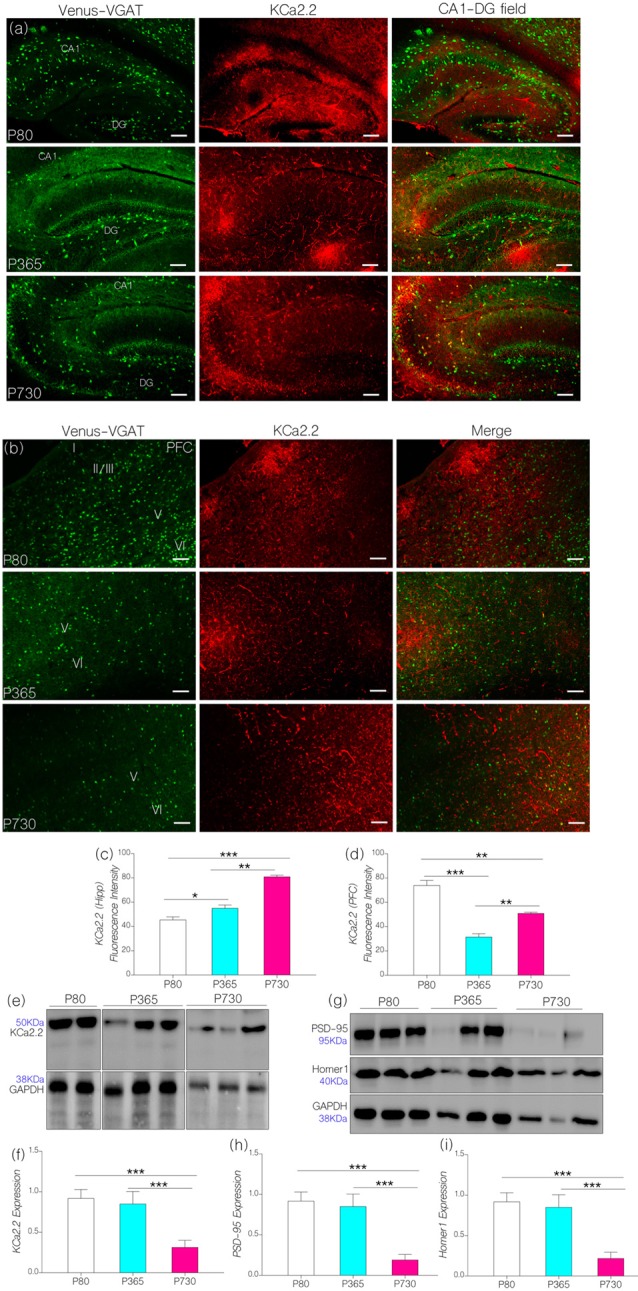
**(A)** Representative confocal images showing a KCa2.2 expression pattern in the hippocampus (scale bar = 50 μm). (**B)** Confocal images showing the cortical distribution of KCa2.2 in the PFC (scale bar = 50 μm). **(C)** Bar chart depicting a statistical comparison for KCa2.2 expression in the hippocampus. An age-dependent increase in hippocampal KCa2.2 expression was recorded at P365 (*p* < 0.05) and P730 (*p* < 0.001) when compared with P80. **(D)** Bar chart showing a significant decrease in cortical KCa2.2 for P365 (*p* < 0.001) and P730 mice (*p* < 0.01); vs. P80 expression level. **(E,F)** Quantitative western blots showing a decreased KCa2.2 expression in total brain lysate at P730 when compared with P80 or P365 (*p* < 0.001). **(G–I)** Quantitative western blots showing a significant decrease in post-synaptic markers in P730 total brain lysate. PSD-95 (*p* < 0.001) and Homer-1 expression (*p* < 0.001) reduced significantly for the P730 group when compared with P80 or P365 mice. *p < 0.05, **p < 0.01, ***p < 0.001.

In KCa2.2 immunolabeled sections, we found that KCa2.2 expression varied in the hippocampus and cortex (Figures 5A,B). In the hippocampus, normalized fluorescence intensity of immunolabeled KCa2.2 (expression) increased significantly at P365 (*p* < 0.05) and P730 (*p* < 0.001) when compared with P80 (Figure 5C). Additionally, we observed an age-linked increase in hippocampal KCa2.2 expression when comparing P365 with P80 (*p* < 0.05), and P730 vs. P365 (*p* < 0.01; Figure 5C). The outcome for the hippocampal expression of IGF-1R, MAPK, CaMKIIα and KCa2.2 support our hypothesis. As such a change in hippocampal IGF-1R was associated with increased MAPK phosphorylation (Figure 3B), depleted CaMKIIα (Figure 4C) and upregulated KCa2.2 expression (Figure 5C).

Surprisingly, decreased cortical CaMKIIα (Figure 4D) and increased MAPK/ErK (Figure 3C) was associated with reduced KCa2.2 expression for the P365 mice (*p* < 0.001); when compared with P80 mice (Figure 5D). However, there was a significant increase in cortical KCa2.2 expression at P730 (*p* < 0.01) when compared with P365 expression levels. This outcome agrees partially with our hypothesis, since the mPFC exhibits a distinct pattern of KCa2.2 expression when compared with the hippocampus in aged mice. In the mPFC, CaMKIIα expression was significantly sustained at P365 when compared with P730 (*p* < 0.05; Figures 4E,F). Conversely, the expression of phosphorylated CaMKIIα was significantly lower in P365 brain lysate when compared with P730 expression (*p* < 0.01; Figures 4G,H). Pertaining to CaMKIIα regulation of KCa2.2 function, we deduced that a decrease in cortical CaMKIIα expression at P365 was not sufficient to cause upregulation of KCa2.2 in the mPFC. Rather, a decreased expression together with an increased percentage phosphorylation (inactivation) of CaMKIIα might have contributed to upregulation of cortical KCa2.2 at P730 (Figure 5D). Taken together, we infer that decreased expression and increased phosphorylation of CaMKIIα may be associated with dysregulation of KCa2.2 in the CA1 and mPFC.

In subsequent analysis, we found that neural KCa2.2 did not significantly change at P365 in whole brain lysate (Figures 5E,F). However, it is important to note that regional variations may occur, as observed for the hippocampus and cortex through immunohistochemical methods. As such, KCa2.2 expression increased in the hippocampus but decreased in the mPFC at P365 (Figures 5C,D) when assessed through immunohistochemistry. At P730, there was a significant decrease in whole brain KCa2.2 expression (Figure 5F; *p* < 0.001) although hippocampal KCa2.2 (confocal) expression remained significantly higher when compared with P80 (*p* < 0.001) and P365 (*p* < 0.01). In order to ascertain a change in post-synaptic profile, we evaluated the expression of post-synaptic structural proteins that are closely related to synaptic function, KCa2.2 and CaMKIIα expression at PSDs densities. In support of our results, there were significant changes in the expression of PSD-95 and Homer1 in whole brain lysate of aged (P730) animals when compared with P80 and P365 expression (*p* < 0.001; Figures 5G–I).

### Synaptic Excitatory and Inhibitory Transport

**Figure 6:**
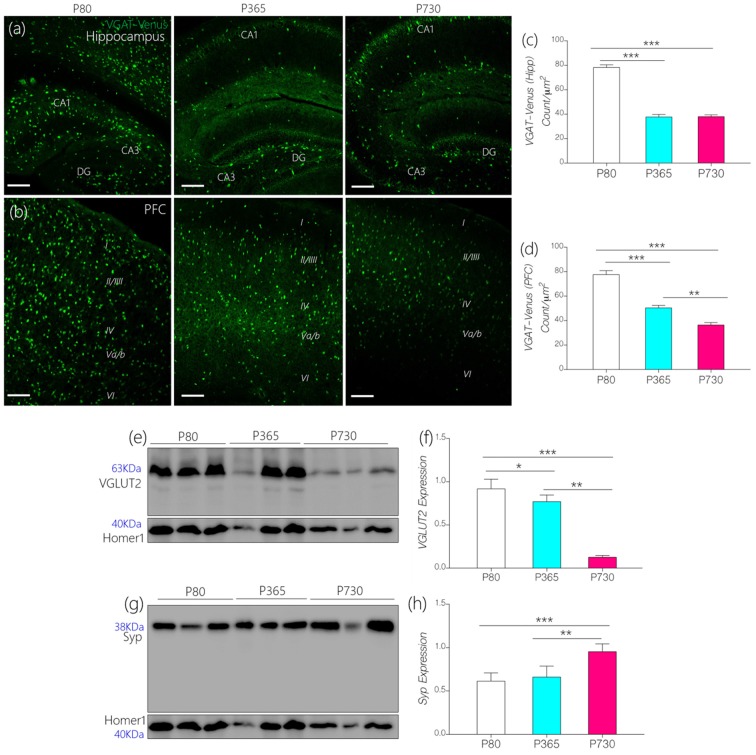
**(A,B)** Representative confocal images showing age-dependent changes in the distribution of inhibitory neurons in the hippocampus and prefrontal cortex (PFC; scale bar = 50 μm). **(C,D)** Bar chart depicting a statistical change in PFC and hippocampal inhibitory neurons (VGAT-Venus) cell count. A significant decrease in inhibitory neurons was recorded for the P365 and P730 hippocampus and cortex (*p* < 0.001, *p* < 0.001) when compared with P80. **(E,F)** Quantitative immunoblots showing a significant increase in synaptophysin expression in whole brain lysate of P730 (*p* < 0.001) mice when compared with P80 (*p* < 0.001) and P365 (*p* < 0.01). **(G,H)** Quantitative immunoblots showing a significant decrease in neural vesicular glutamate transporter 2 (VGLUT2) expression at P365 (*p* < 0.05) and P730 (*p* < 0.001) when compared with P80 expression. *p < 0.05, **p < 0.01, ***p < 0.001.

Since IGF-1R is involved in the modulation of presynaptic function (Gazit et al., 2016), we compared age-linked changes in the expression of IGF-1R and presynaptic proteins associated with vesicle and neurotransmitter transport. Furthermore, we highlighted possible links between dysregulated IGF-1R- CaMKIIα-KCa2.2 function and excitatory/inhibitory neurotransmitter transporter protein expression in the CA1 and mPFC regions. In addition to age-linked decreases in IGF-1R, there was a significant decrease in the count of inhibitory GABAergic and Glycinergic neurons expressing VGAT (Figures 6A–D). In the hippocampus (CA1-DG field), the count of VGAT-Venus neurons decreased at P365 (*p* < 0.001) and P730 (*p* < 0.001) when compared with P80 count (Figures 6A,C). Likewise, VGAT-Venus neuron count decreased in the mPFC of P365 and P730 mice when compared with P80 scores (Figures 6B,D). Equally, there was a significant loss of VGLUT2 in whole brain lysate of aged mice (Figures 6E,F). This suggests a significant change in presynaptic morphology; similar to changes in post-synaptic protein expression described previously (PSD-95 and Homer1; Figures 5G–I). Interestingly, synaptophysin, a presynaptic protein, increased in total brain lysate at P730 when compared with P80 (Figures 6E,F; *p* < 0.001) and P365 expression (*p* < 0.01). Although IGF-1R is known to mediate the synaptic activity of synaptophysin (Gazit et al., 2016), our results suggest an inverse relationship for this interaction. As such, a decrease in neural IGF-1R was accompanied by an increase in synaptophysin expression. This may represent a compensatory mechanism for the loss of synaptic function in the aging brain. Based on these outcomes, we deduced that loss of IGF-1R signaling in the aged brain may be linked to depletion of post-synaptic proteins, dysregulation of IGF-R-linked presynaptic neurotransmitter transport, and synaptophysin activity.

## Discussion

The role of IGF-1 and IGF-1R have been extensively described in the pathophysiology of age-related brain disorders and developmental synaptic dysfunction (van Dam and Aleman, 2004; Chiu and Cline, 2010; Fernandez and Torres-Alemán, 2012; Dyer et al., 2016; Reim and Schmeisser, 2017; Wrigley et al., 2017). The outcome of this study demonstrates that age-related changes in IGF-1/IGF-1R activity may be associated with dysregulated synaptic MAPK/ErK and CaMKIIα function. We found that an age-related decrease in IGF-1/IGF-1R expression was associated with reduction of neural CaMKIIα expression and increased MAPK/ErK phosphorylation in the brain. Based on our hypothesis (Figure 1), the physiological implication of these outcome may involve increased activity of small ion conductance channels (KCa2.2) at hippocampal PSDs, and decreased expression in the mPFC of aged mice. Increased KCa2.2 expression in the hippocampus may occur due to reduced CaMKIIα-dependent KCa2.2 inhibition, increased CaMKIIα phosphorylation (inactivation) by p-MAPK/ErK, and upregulated phosphorylation (activation) of KCa2.2 by p-MAPK/ErK. Taken together, our results suggest that age-linked changes in IGF-1R signaling may alter synaptic KCa2.2 regulation by disrupting the balance of regulatory synaptic proteins, CaMKIIα and MAPK/ErK.

### IGF-1/IGF-1R-Linked Alteration in Synaptic Kinases

IGF-1R is activated by endogenous IGF-1 (Dyer et al., 2016; Gazit et al., 2016). This interaction contributes to the regulation of presynaptic Ca^2+^ signaling and Ca^2+^ release from the mitochondria (Gazit et al., 2016), N-type, and L-type calcium channels (Blair and Marshall, 1997). Although, synaptic function (LTP) involves a synchronous oscillation of Ca^2+^ and K^+^ ions (Bacci et al., 1999; Power et al., 2002), the effect of IGF-1/IGF-1R signaling on the activity of calcium-dependent potassium channels (KCa2.2) is poorly understood. Furthermore, how an age-dependent change in IGF-1/IGF-1R signaling contributes to dysregulation of synaptic KCa2.2 function in the aged brain has yet to be investigated. In this study, we described some of the possible pathways through which a change in IGF-1R signaling can alter synaptic KCa2.2 activity in the aging cortex and hippocampus.

### Alterations in Synaptic Kinases and KCa2.2 Expression

Our results indicate that a decrease in IGF-1 and IGF-1R with age was associated with a significant change in the expression and phosphorylation of synaptic kinases involved in synaptic function. Hippocampal and prefrontal cortical IGF-1R expression decreased by middle age as seen in P365 mice (Figures 2C,D). However, depletion of brain IGF-1 occurred much later in P730 brain lysate (Figures 2G,H). To test our hypothesis, we evaluated the significance of age-linked IGF-1/IGF-1R alteration on the relative expression of MAPK/ErK and CaMKIIα in the hippocampus and mPFC of mice. In addition to acting downstream of IGF-1R (Chiu and Cline, 2010; Deak and Sonntag, 2012; Dyer et al., 2016), both proteins (i.e., MAPK/ErK and CaMKIIα) are involved in the regulation of neurotransmitter receptors and ion channels at synapses (Giovannini et al., 2001; Tsui et al., 2005). Moreover, previous studies have described co-localization of MAPK/ErK and CaMKIIα at post-synaptic sites (Giovannini et al., 2001; Tsui et al., 2005; Hammond et al., 2006). Since IGF-1R regulates MAPK/ErK and CaMKIIα through the Ras/Raf/ErK pathway, a change in IGF-1R signaling may alter the synaptic activity of MAPK/ErK and CaMKIIα. In aging, a decline in IGF-1R may cause an increase in MAPK phosphorylation. Thus, an increase in activated p-MAPK/ErK, can facilitate phosphorylation (inactivation) of CaMKIIα thereby disrupting synaptic function (Ferrer et al., 2001; Giovannini et al., 2001; Hoofnagle et al., 2004).

Age-dependent changes in IGF-1/IGF-1R are associated with dysregulation of MAPK/ErK and CaMKIIα expression (Figures 3, 4). MAPK/ErK and CaMKIIα modulates NMDAR-linked calcium currents (Hinds et al., 2003; Mao et al., 2014) and calcium-activated potassium channels (KCa2.2) during LTP (Giovannini et al., 2001; Hammond et al., 2006; Lin et al., 2010). Therefore, we examined the differential expression of KCa2.2 in the hippocampus and cortex of aged mice, characterized by a decrease in IGF-1/IGF-1R and altered MAPK/CaMKIIα expression (Figure 5). Our results show that an increase in p-MAPK/ErK, and decreased CaMKIIα was associated with upregulated KCa2.2 expression in the hippocampus of aged mice. Although previous studies show a change in Ca^2+^ currents relative to IGF-1R function (Blair and Marshall, 1997; Gazit et al., 2016), here we propose a possible mechanism through which IGF-1R might alter K^*+*^ current through the regulation of substrates that modulate synaptic KCa2.2 function in aging.

The physiological implication of increased neural KCa2.2 activity has been described previously by Hammond et al. (2006). They demonstrate that an increase in KCa2.2 activity abolishes synaptic potentials and reduced memory function in mice (Hammond et al., 2006; Maingret et al., 2008; Lin et al., 2010). In separate studies, changes in the expression of KCa2.2 in young mice precipitated a decline in memory formation and retrieval (Stackman et al., 2002; Hammond et al., 2006). Evidently, the expression and activity of K*Ca2.2* represents a crucial part of synaptic regulation and LTP (Disterhoft and Oh, 2006, 2007). Expression of KCa2.2 and other post-synaptic proteins—PSD-95 and Homer1–show significant declines with age in the brain. Thus, differential hippocampal and cortical KCa2.2 expression suggest adaptive physiological changes in normal synaptic aging. Our results suggest that decreased CaMKIIα, increased CaMKIIα phosphorylation, and increased MAPK/ErK phosphorylation are possible causes of synaptic KCa2.2 dysregulation that may be linked to decreased IGF-1R signaling in the normal aging brain.

### Neurotransmission

In addition to a change in KCa2.2 expression and post-synaptic proteins (Homer1 and PSD-95), we found a significant change in presynaptic protein expression in the aged brain. While previous studies have shown the role of IGF-1R in the presynaptic activity of synaptophysin (Gazit et al., 2016), here we showed an inverse relationship between IGF-1R and synaptophysin expression in the aged (P730) brain. Our results indicate that a decrease in IGF-1R was associated with a significant increase in synaptophysin expression with age (Figures 6G,H). Conversely, there was a decrease in the expression of Venus in VGAT-positive puncta (confocal) and VGLUT2 (immunoblots) in the brain of aged mice (P730). From these outcomes, we deduced that an increased p-MAPK/ErK can increase the activity of synaptophysin independent of neurotransmitter transport. Yokomaku et al. (2003) support this proposition by showing that MAPK signaling inhibitors suppressed synaptophysin function in cultured neurons. Synaptophysin expression and activity were rescued by estradiol-mediated MAPK/ErK increase *in vitro* (Yokomaku et al., 2003). Since VGAT and VGLUT2 exist in the presynaptic area (Zander et al., 2010), it is logical to speculate that a change in CaMKIIα control of KCa2.2 function may alter the synaptic expression and activity of VGAT and VGLUT2 (Trimmer, 2015), although the mechanism remains to be resolved. As such, selective activation of IGF-1R and inhibition of KCa2.2 function are possible intervention methods for attenuating CaMKIIα loss and synaptic dysfunction in aging.

## Conclusion

Taken together, the outcome of this study showed that neural IGF-1/IGF-1R expression is reduced with age in the hippocampus and cortex. IGF-1/IGF-1R depletion is linked to increased neural MAPK/ErK phosphorylation and CaMKIIα depletion in the aged brain. We showed that loss of IGF-1/IGF-1R was also associated with a change in the synaptic expression of KCa2.2; especially in the hippocampus of aged mice. Ultimately, this might lead to a decline in presynaptic neurotransmitter function and loss of post-synaptic proteins.

## Author Contributions

OMO, CCL and JF conducted specific aspects of the research. CCL, JF and RGG supervised the manuscript write up and analysis of data. JP and OMO completed manuscript write up and presentation of data and conducted additional experiments for protein analysis.

## Conflict of Interest Statement

The authors declare that the research was conducted in the absence of any commercial or financial relationships that could be construed as a potential conflict of interest.
